# Genomic Redistribution of Metal-Response Transcription Factor-1 (MTF-1) in Cadmium Resistant Cells

**DOI:** 10.3390/cells12060953

**Published:** 2023-03-21

**Authors:** Gregory M. Wright, Joshua C. Black

**Affiliations:** Department of Pharmacology, University of Colorado Anschutz Medical Campus, Aurora, CO 80045, USA

**Keywords:** MTF-1, cadmium, metal response

## Abstract

(1) Background: Metal homeostasis is an important part of cellular programs and is disrupted when cells are exposed to carcinogenic heavy metals. Metal response is mediated by the metal response element transcription factor MTF-1. However, where MTF-1 binds and how that binding changes in response to heavy metals, such as cadmium, remains unknown. (2) Methods: To investigate the effects of prolonged cadmium exposure on the genomic distribution of MTF-1, we performed MTF-1 CUT&RUN, RNA-seq and ATAC-seq on control and cadmium-resistant cells. (3) Results: Changes in MTF-1 binding primarily occur distal to the transcription start sight. Newly occupied MTF-1 sites are enriched for FOS/JUN DNA binding motifs, while regions that lose MTF-1 binding in cadmium are enriched for the FOX transcription factor family member DNA binding sites. (4) Conclusions: Relocalization of MTF-1 to new genomic loci does not alter the accessibility of these locations. Our results support a model whereby MTF-1 is relocalized to accessible FOS/JUN-bound genomic locations in response to cadmium.

## 1. Introduction

Metal homeostasis regulates the cellular levels of essential metals important for the functioning of enzymes, transcription factors, and electron transport, thus regulating all biological processes within the cell. Other metals without endogenous sources, including cadmium, mercury and lead are toxic, carcinogenic, and disrupt essential metal homeostasis [1]. The cellular metal responses to essential and toxic metals are controlled by metal-responsive transcription factors that act through metal response element DNA sequences. Metal response elements (MREs) were initially identified as DNA sequence motifs adjacent to mouse metallothionein (MT) genes [2,3]. In response to cadmium and zinc, these DNA elements supported increased gene expression of metal-responsive genes [4,5]. Searches for the DNA-binding factor that recognizes the MRE eventually identified the MRE-binding transcription factor-1 or MTF-1 [6]. MTF-1 was revealed to be a zinc finger containing a transcription factor able to activate metal-responsive genes in mice [7]. A human homolog was discovered with high sequence conservation to the mouse MTF-1 [5], which is also conserved in flies, fish, and mammals [8,9]. MTF-1 is essential for normal metal homeostasis and for the cellular response to heavy metals [10]. MTF-1 also contributes to the cellular response to stress, including hypoxia and oxidative stress [11]. MTF-1 can be activated directly by zinc or zinc released from MT in response to heavy metal loads or oxidative stress [12]. Under normal conditions, MTF-1 is found in both the nucleus and cytoplasm, but the concentration shifts to the nucleus upon induction of cellular stress, including excess metals and oxidative stress [13].

MTF-1 can recognize MREs when bound to metal; regular binding of MTF-1 to cellular zinc modulates MTF-1 activity [14,15]. Under normal conditions, cellular zinc is bound to MT proteins. When the cell is exposed to excess metals, such as cadmium, the MT-bound zinc is replaced by cadmium, increasing cellular zinc levels and increasing the activity of MTF-1 [16]. However, it has been shown that cadmium-induced MTF-1 activity does not utilize the standard MRE binding motif [14]. This suggests that cadmium-induced MTF-1 exhibits alternative DNA binding compared to other metals. It remains unclear how MTF-1 is redistributed throughout the genome in response to cadmium.

MTF-1 levels are elevated in tumors including breast, lung and cervical tumors [17]. Cadmium levels in breast tissue correlate with a higher risk of breast cancer [18], while increased levels of cadmium are found in breast cancer tissue compared to adjacent normal tissue [19]. Additionally, it has been shown that cadmium can transform normal breast cells [20]. Thus, it is important to understand the genes directly bound by MTF-1 and how these change during metal stress as they may reveal novel insights about cadmium-induced transformation and oncogenesis.

In this study, we analyzed how MTF-1 binding changes in response to prolonged cadmium stress. MTF-1 binding changes occurred primarily distal to gene promoters. Cadmium induced modest changes in gene expression, some of which were directly regulated through changes in the binding of MTF-1. MTF-1 binding was enriched at locations with DNA motifs for Fos and Jun transcription factor families and lost from locations with DNA motifs for Forkhead-box (FOX) gene family. Through RNA-seq, we demonstrate that cadmium treatment upregulates the metal and stress response genes, but also downregulates genes related to cell migration and differentiation. Finally, we demonstrate that chromatin accessibility is unchanged at regions where MTF-1 is relocalized, suggesting that chromatin accessibility is determined prior to MTF-1 relocalization.

## 2. Materials and Methods

### 2.1. Cell Culture

The MDA-MB-231 female breast cancer cell line was acquired from the ATCC and was maintained at 37 °C in Dulbecco’s Modified Essential Medium (DMEM) with 10% FB essence (VWR), 1% penicillin/streptomycin, and L-glutamine. Cadmium-exposed cell lines were selected by serial passaging in a medium containing CdSO_4_. The CdSO_4_ concentration started at 5 μM and was increased to 10 μM [21,22,23] after 10 passages, cells were grown in 10 μM for at least 10 passages before use in experiments. Both CdSO_4_ and CdCl_2_ have been used to investigate the effects of cadmium on cells with similar effects. CdSO_4_ has been used in animal studies, in rodent cells and breast cancer studies [24,25,26]. Mycoplasma presence was periodically analyzed through DAPI staining and imaging of growing cells. Cell cultures were maintained free of mycoplasma.

### 2.2. Total RNA Extraction

Total RNA was extracted from the control and cadmium cells using the miRNeasy Mini Kit (Qiagen, Valencia, CA, USA) following the manufacturer’s protocol including the optional DNase digest. RNA concentration and purity were determined with a ThermoScientific Nanodrop 2000 Spectrophotometer (Waltham, MA, USA).

### 2.3. CUT&RUN

Cleavage Under Targets and Release Using Nuclease (CUT&RUN) experiments were carried out following Epicypher CUT&RUN protocol (version 1.6, August 2020) with minor modifications. Briefly, nuclei from 5 × 10^5^ cells were isolated with wash buffer (20 mM HEPES-KOH, pH 7.5, 150 mM NaCl, 0.5 mM Spermidine, and 1× protease inhibitor cocktail (Sigma, St. Louis, MO, USA), captured with Concanavalin A conjugated paramagnetic beads (Bangs Laboratory Inc., Fishers, IN, USA) and incubated while nutating with 1 µL primary antibody for MTF-1 (Santa Cruz H-6 sc-365090) in antibody buffer (20 mM HEPES-KOH, pH 7.5, 150 mM NaCl, 0.5 mM Spermidine, 0.02% digitonin, 2 mM EDTA and 1× protease inhibitor cocktail (Sigma, St. Louis, MO, USA) overnight. After washing with digitonin buffer (20 mM HEPES-KOH, pH 7.5, 150 mM NaCl, 0.5 mM Spermidine, 0.02% digitonin and 1× protease inhibitor cocktails from Sigma), pAG-MNase (Epicypher Inc., Durham, NC, USA) was added at a 1:20 ratio and incubated for 10 min at room temperature (RT). The nuclei were washed again and placed on ice. To activate pAG-MNase, CaCl_2_ was added to a final concentration of 2 mM. The reaction was incubated for 2 h while nutating at 4 °C and stopped by the addition of STOP buffer (340 mM NaCl, 20 mM EDTA, 4 mM EGTA, 50 mg/mL RNase A and 40 mg/mL glycogen). The protein–DNA complex was released by incubating for 10 min at 37 °C. DNA was extracted using a Qiagen Minelute PCR purification kit. Purified DNA was used for library preparation.

### 2.4. CUT&RUN Library Preparation, Sequencing and Data Processing

CUT&RUN libraries were prepared using the Illumina NEBNext Ultra II ChIP-Seq sample kit according to the manufacturer’s protocol. Libraries were validated using the Agilent Technologies 2100 Bioanalyzer (Santa Clara, CA, USA). Libraries were sequenced by the University of Colorado Cancer Center Genomics and Microarray Core on Illumina NovaSEQ6000 sequencer (San Diego, CA, USA) as paired-end 151 × 8 × 8 × 151. CUT&RUN FASTQs were adaptor trimmed using BBtools bbduk (v.38.87), using supplied adaptor reference file adapters.fa with the parameters ktrim = r k = 23 mink = 11 hdist = 1 tpe tbo, then aligned to hg38 genome using Bowtie2 (v.2.3.4.1). Sam files were transformed into bam files and biological replicates were merged using samtools merge (version 1.8). CUT&RUN alignment coverage bigwig files were created with deeptools bamCoverage (v.3.1.3) using CPM with 50 bp binning. Sequencing files, coverage bigwigs and called peaks are deposited in the GEO archive under accession number: GSE222587.

### 2.5. Peak Calling

CUT&RUN peak calling was performed using Epic2 v.0.0.50, using default parameters with merged bam files (see ChIP-seq Preparation, Sequencing and Data Processing) as -reatment (MTF-1) using -genome hg38 for both the control cells and 10 µM cadmium cells. Peaks unique to control or cadmium-treated cells were identified using bedtools intersect (v.2.26.0) using parameter -v to only report peaks with no overlap. Common peaks between samples were identified using bedtools intersect (v.2.26.0) using parameter -wa to identify overlapping peaks in each sample with at least 1 base overlap, followed by bedtools merge (v.2.26.0) to create a single list of conserved overlapping peaks combining all overlapping peaks to a single output peak. A randomized control peak set with the same number of peaks and equivalent distribution of peak widths was created using bedtools shuffle (v.2.26.0) with default settings using combined peak sets (control peaks, cadmium peaks and conserved peaks) as the input and GRCh38 as genome.

### 2.6. Analysis of Motif Enrichment

Motif enrichment was performed using MEME suite AME (v.5.4.0). Fasta for MTF-1 peaks were generated using bedtools getfasta (v.2.26.0) for 500 bp of sequence surrounding the peak center from GRCh38 reference genome (NCBI). Analysis was run on each set of unique peaks as input with consensus control peaks as -control (see Peak Calling) using default parameters. JASPAR 2022 CORE vertebrate’s non-redundant set of motifs [27] as MEME files was used in this analysis.

### 2.7. ATAC-Seq Library Preparation, Sequencing and Data Processing

ATAC-seq libraries were prepared using Active Motif ATAC-seq kit (cat. no. 53150) following the manufacturer’s instructions. Libraries were sequenced by Novogene Co. on Illumina Hiseq 4000 (Sacramento, CA, USA) as paired-end reads. ATAC-seq FASTQ files were run using ENCODE ATAC-seq pipeline 1.8.0. Bam files generated for replicates were merged using samtools merge (version 1.8). Alignment coverage bigwig files were created with deeptools bamCoverage (v.3.1.3) using CPM with 50 bp binning. Sequencing files and coverage bigwigs are deposited in the GEO archive under accession number: GSE216496.

### 2.8. RNA-Seq Library Preparation, Sequencing and Data Processing

Purified total RNA samples (see Total RNA Extraction) were sent to the University of Colorado Cancer Center Genomics and Microarray Core, which prepared the libraries and sequenced them using an Illumina NovaSEQ6000 with paired-end 150 bp reads. RNA-seq FASTQs were adaptor trimmed using BBtools bbduk (v.38.87) using supplied adaptor reference file adapters.fa with parameters ktrim = r k = 23 mink = 11 hdist = 1 tpe tbo then aligned to hg38 genome using hisat2 (v.2.1.0). Sequence reads were assigned to genomic features using featureCounts (v.2.0.1) using ensemble_v105_hg38.gtf. Differential expression analysis was performed using DESeq2 (v.1.34.0) on Rstudio (v.1.4.1717). Sequencing files and featureCounts files are deposited in the GEO archive under accession number: GSE220803.

### 2.9. Gene Set Enrichment Analysis

Gene set enrichment analysis was performed using GSEA (v.4.3.1) on differentially expressed genes identified with DESeq2 (See RNA-seq library preparation) and gene sets obtained from https://www.gsea-msigdb.org/ (accessed on 5 January 2023) using default settings.

### 2.10. Gene Ontology

Gene ontology analysis was performed using Geneontology.org. The list of differentially expressed genes (either upregulated or downregulated) was input for biological processes or cellular components using the default parameters and results for FDR *p* < 0.05. The lowest child GO nodes from the results were chosen for this analysis.

### 2.11. Ingenuity Pathway Analysis

The significantly differentially expressed genes were analyzed using Upstream Regulatory Analysis in Ingenuity Pathway Analysis (IPA) (QIAGEN Inc., Valencia, CA, USA). Significantly enriched upstream regulators are presented in Table S2. Note that MTF-1 is not recognized as an upstream regulator in IPA.

## 3. Results

### 3.1. Cadmium Promotes Relocalization of MTF-1

**Figure 1:**
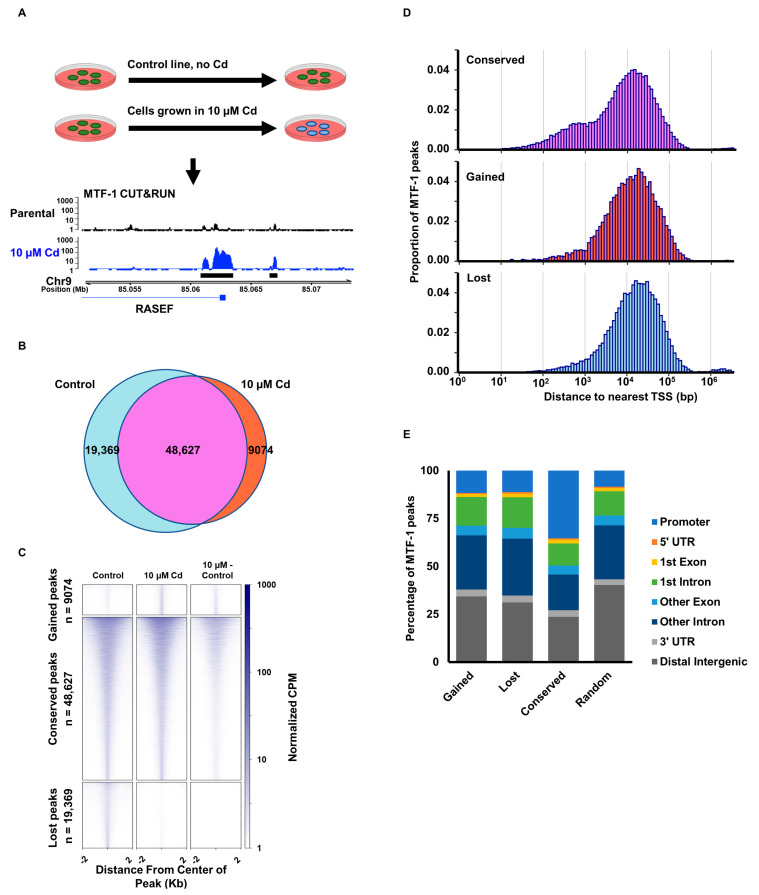
Relocalization of MTF-1 in response to cadmium. (**A**) MDA-MB-231 cells were treated chronically with cadmium to develop a cadmium-exposed cell line. CUT&RUN for metal factor transcription factor MTF-1 was performed on both cadmium-exposed cells and matching untreated control cells. Tracks show normalized MTF-1 signal in control and cadmium-treated cells. Black bars below represent peaks called in that sample. (**B**) Venn diagram representing the overlap among MTF-1 CUT&RUN peaks for control and cadmium-exposed lines shows distribution of peaks conserved between samples, peaks gained from cadmium treatment, and peaks lost from cadmium treatment. (**C**) Heatmaps of the normalized signal of MTF-1 around the center of MTF-1 peaks for control cells, cadmium-exposed cells, and the difference between cadmium and control cells (Cadmium-Control). Heatmaps are separated for peak groupings identified in (**B**). (**D**) CUT&RUN peaks were associated with the closest gene transcription start site (TSS), histograms show distribution of the distance from the center of the peak to the closest TSS for groups of peaks defined in (**B**). (**E**) Genomic annotations for peak groups defined in (**B**) compared to the distribution of randomized genomic locations of all called peaks.

Metal homeostasis is important for regulating all biological processes in the cell. It is unclear how the disruption of metal homeostasis through prolonged metal stress alters the regulation by the metal response element transcription factor, MTF-1. To investigate this, we treated MDA-MB-231 cells chronically with 10 µM cadmium to establish a prolonged cadmium exposure at a concentration known to induce lipid peroxidation, ROS production and DNA damage in breast cancer cells [28,29,30]. We analyzed the binding of MTF-1 using Cleavage Under Targets and Release Using Nuclease (CUT&RUN) [31] (Figure 1A). MTF-1 peak calling (Figure 1B) identified three classes of peaks: (1) peaks present in control cells that disappeared with cadmium treatment, (2) peaks present in the cadmium-treated cells, but not present in the control cells, and (3) peaks conserved in both cadmium and control cells. The conserved peaks group consists of overlapping peaks from both the control and cadmium cells, merged to create a consensus set of peaks. MTF-1 signal intensity surrounding the called peaks in all 3 groups is highest directly at the center of the called peaks (Figure 1C) and existed in a broad distribution of peak width and intensity through the 77,070 identified binding sites. Next, we associated each peak with the closest gene transcription start site (TSS). Analysis of the distance to the closest TSS shows that the conserved peaks have a larger proportion of peaks within 1 Kb of the nearest gene TSS compared to peaks unique to either the control or cadmium-treated cells (Figure 1D).

Consistent with this observation, ChIPseeker analysis of peak distributions demonstrates that the conserved peaks have more than double the proportion of peaks assigned to the promoter region (35.4%) compared to unique peaks from control and cadmium-treated cells or a randomized set of control peaks (11.7%, 11.3%, and 8.5%, respectively) (Figure 1E). Overall, this suggests that cadmium exposure alters MTF-1 binding throughout the genome and that most of these changes occur distal to the transcription start site of genes, likely reflecting changes at distal enhancer elements.

### 3.2. Cadmium Relocalizes MTF-1 to FOS/JUN Motifs

**Figure 2:**
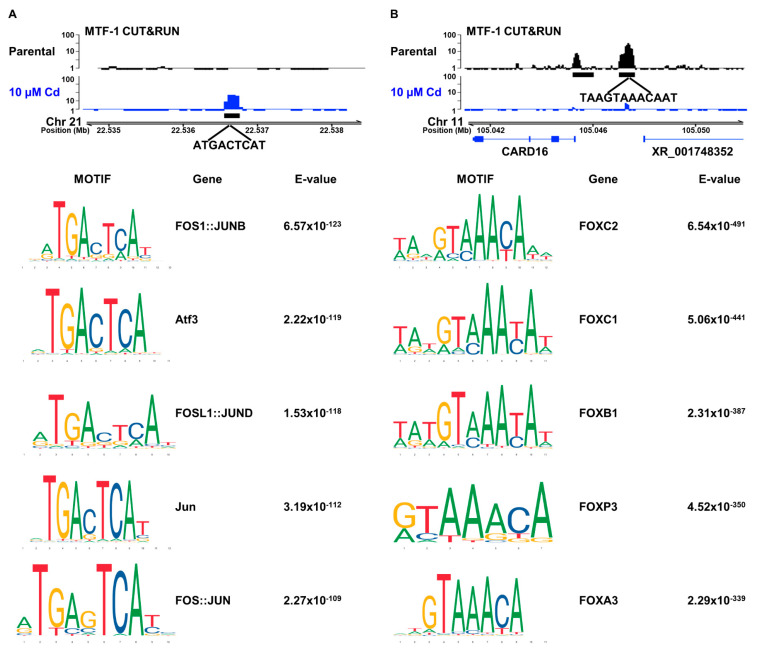
Cadmium induces MTF-1 binding preferentially at FOS/JUN motifs. Top results for Meme suite AME motif enrichment of JASPAR 2022 core vertebrate non-redundant motif library for MTF-1 peaks (**A**) gained from cadmium treatment and (**B**) lost from cadmium treatment. Both peak groups are compared to conserved MTF-1 peaks to identify motif enrichment specific to these subsets of peaks. Gene motifs were obtained from JASPAR (see methods) and e-value is the adjusted p-value calculated by AME.

We performed motif analysis for DNA sequences surrounding the MTF-1 peaks gained and lost from cadmium treatment. We extracted the DNA sequence 250 bp up and downstream of the peak center. This 500 bp sequence for all 9074 gained peaks and 19,369 lost peaks were analyzed using the 48,627 conserved peaks as a control for the presence of 841 DNA sequence motifs from the JASPAR 2022 Core vertebrates non-redundant motif collection [27] and Multiple Expectation maximization for Motif Elicitation (MEME) suite Analysis of Motif Enrichment (AME) software. We found MTF-1 locations newly bound in cadmium-treated cells were enriched for FOS/JUN family member motifs (Figure 2A). FOS/JUN are members of the AP-1 transcription factor which is associated with cellular growth, cellular proliferation, and apoptosis in response to toxic metal exposure [32]. Locations that lost MTF-1 binding in response to cadmium (i.e., peaks unique to control cells) were enriched for Forkhead box (FOX) transcription factor family member sequence motifs (Figure 2B).

JASPAR reports a motif of TGCACACG, which is derived from high-throughput systematic evolution of ligands by exponential enrichment (HT-SELEX) with position weight matrix (PWM) analysis [33,34]. This does not appear to be a bona fide MTF-1 motif and is not selected for in other work to identify MTF-1 binding motifs [35]. Consistent with the model that this is not an MTF-1 motif, we did not observe an enrichment of this motif in our data. This is also consistent with reports that MTF-1 did not bind to consensus MREs in the presence of cadmium, but did in the presence of excess zinc [14]. Consistent with the Schaffner group’s results, we do not find enrichment for a particular de novo motif associated with cadmium-activated MTF-1. Instead, MTF-1 appears to function with FOS/JUN family motifs in response to cadmium and work with FOX family motifs in control cells (Figure 2). Overall, this suggests that MTF-1 may bind to DNA differently in the presence of cadmium compared to other metals, or that alternative pathways modify expression levels within the cell from cadmium.

### 3.3. Differential Gene Expression Resulting from Cadmium Exposure

**Figure 3:**
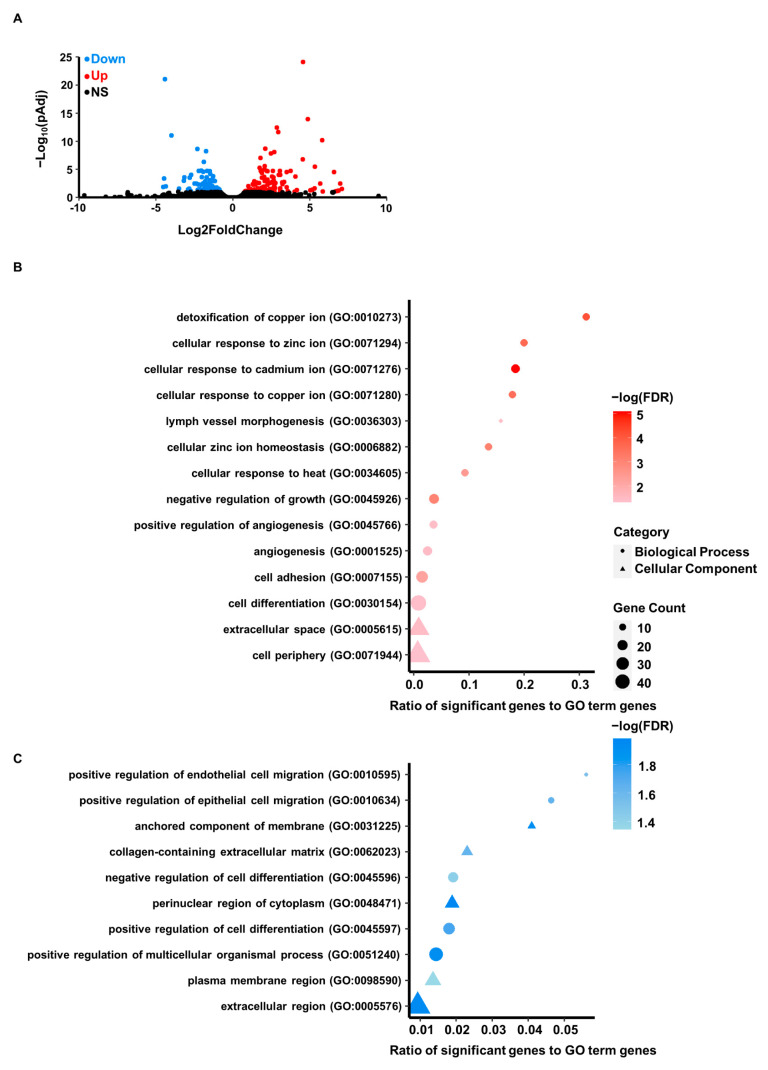
Differential gene expression resulting from cadmium exposure. (**A**) RNA-seq was performed on cadmium exposed MDA-MB-231 cells. Volcano plot shows genes defined as upregulated (red), downregulated (blue), and not significantly changed (black). Gene ontology was performed on (**B**) 119 upregulated genes and (**C**) 106 downregulated genes. Over-represented GO terms are shown for biological process (BP, circle) or cellular component (CC, triangle) and are sorted by gene ratio (ratio of number of genes associated with the process to total genes in the GO term). The icon size indicates the number of genes associated with the process and the icon color indicates the significance of the enrichment (-log10(FDR-corrected *p*-values). FDR-corrected *p*-values are calculated using Fisher’s Exact test.

Cellular cadmium exposure alters MTF-1 binding, which should facilitate gene expression changes related to metal homeostasis. To investigate the effect of prolonged cadmium on gene expression, we performed RNA sequencing (RNA-Seq). Differential gene expression analysis identified 119 upregulated genes and 106 downregulated genes in cadmium-treated cells (Figure 3A and Table S1). To determine what pathways were altered in response to cadmium, we performed gene ontology (GO) analysis of the differentially expressed genes (upregulated Figure 3B; downregulated Figure 3C). The GO terms associated with downregulated genes represented biological processes related to cell migration and differentiation. Additionally, downregulated genes were associated with cellular component GO terms for extracellular regions, cytoplasm, and various membranes. As expected, the GO terms associated with upregulated genes were strongly represented by metal response, metal homeostasis, and stress response groupings. In addition, we performed GSEA analysis on our differential gene expression analysis and found that none of the human hallmark molecular signature gene sets were significantly enriched [36].

The gene sets included in GSEA Human Collection Signature Databases include sets curated for hallmark genes, specific molecular pathways and oncogenic sets suggesting that cadmium was not exacerbating the progression of cancer pathways within our cells.

Alternatively, analysis of the up- and downregulated genes by Ingenuity Pathway Analysis (IPA) Upstream Regulator Analysis identified several transcription factors predicted to be responsible for the observed gene expression changes (Table S2). Importantly, the significantly enriched regulators included FOS/JUN, supporting the identification of relocalization of MTF-1 to FOS/JUN DNA sequence motifs. These results suggest that the relocalization of MTF-1 to FOS/JUN targets facilitates cellular metal stress response.

### 3.4. Cadmium-Induced MTF-1 Binding Does Not Alter Chromatin Accessibility

The changes in MTF-1 localization and gene expression in response to cadmium suggested that changes in chromatin accessibility could be responsible for allowing or occluding MTF-1 binding. To test this hypothesis, we examined the accessibility of chromatin surrounding cadmium-induced differentially expressed genes with an Assay of Transposase-Accessible Chromatin using sequencing (ATAC-seq).

**Figure 4:**
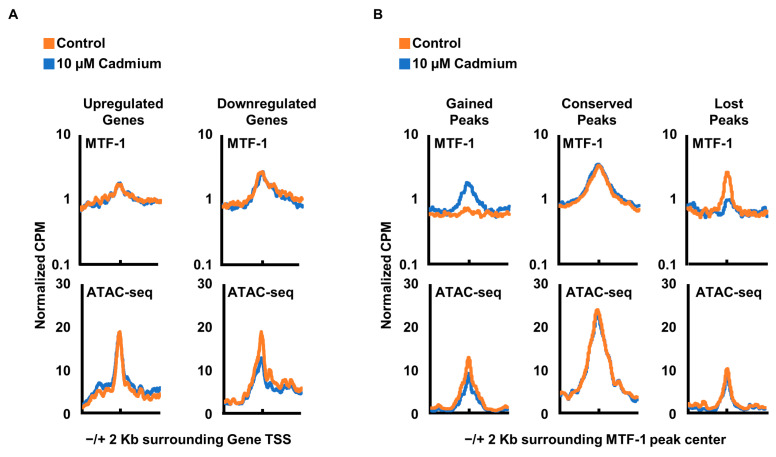
Cadmium-induced MTF-1 binding does not alter chromatin accessibility. (**A**) Normalized signal of MTF-1 and ATAC-seq around the TSS of genes differentially upregulated and downregulated from cadmium exposure for control and cadmium exposed cells. Graphs are separated for TSS groupings between upregulated and downregulated genes. (**B**) Signal for MTF-1 and ATAC-seq around the center of subset of MTF-1 peaks for control line and cadmium exposed line. Graphs are separated for peak groupings identified in (Figure 1B) and only include peaks whose closest gene TSS is a differentially expressed gene.

We compared the MTF1 CUT&RUN and ATAC-seq signals surrounding both upregulated and downregulated genes identified in Figure 3A and observed that the signal peaks for both MTF-1 and ATAC-seq overlap at the transcription start site (TSS; Figure 4A). Next, we analyzed that chromatin accessibility at regions surrounding the MTF-1 binding sites that were increased or lost following cadmium exposure and were adjacent to differentially expressed genes (Figure 4B). While the MTF-1 binding changes for gained and lost peaks, the ATAC-seq signal does not change suggesting that the MTF-1 binding is not controlled by cadmium-induced changes in chromatin accessibility. These results are consistent with a model of increased recruitment of MTF-1 to already accessible chromatin locations.

## 4. Discussion

MTF-1 is a zinc finger transcription factor with a major role in metal homeostasis and metal detoxification. Research has shown that in response to essential metals like zinc, MTF-1 binds to MREs with specific motifs [4,8]. Previous work by the Schaffner group in 2009 suggests that MTF-1 may not localize to specific motifs in response to cadmium [35]. We examined MTF-1 genomic localization in the presence of cadmium and correlated binding with differential gene expression and chromatin accessibility to further understand MTF-1 localization and function in response to cadmium.

We identified classes of MTF-1 peaks, including those gained from cadmium treatment and peaks that disappear from cadmium treatment. The MTF-1 peaks that are gained and lost in cadmium are distal to the gene TSS (Figure 1E). This suggests that MTF-1 response to cadmium is through distal enhancer elements rather than directly through gene promoters. This is supported by the increased association of MTF-1 with FOS/JUN binding motifs in response to cadmium (Figure 2A). The predominant changes in MTF-1 occurring in distal regulatory elements with an enrichment of FOS/JUN motifs are consistent with the strong enrichment for AP-1 family transcription factor binding at distal enhancer elements [37]. We did not observe an enrichment of the MTF-1 motif reported in Jaspar [33], which does not appear to represent a bona fide MTF-1 motif. Wang et. al. identified enrichment of specific sequence motifs for MTF-1 in the presence of copper and zinc but did not observe enrichment for a specific sequence in the presence of cadmium, which is consistent with our genome-wide observations [14]. Tavera-Montanez et. al. showed that MTF-1 bound to a multitude of DNA motifs in developing mice in response to copper, but that cadmium treatment resulted in no specific binding for MTF-1 compared to copper and zinc, which displayed strong enrichment for a sequence-specific MTF-1 binding motif [38]. This supports our findings and shows that the differences in MTF-1 binding induced by cadmium are likely not specific to human cells. However, though no MTF-1-specific motif was identified, we did observe the relocalization of MTF-1 to DNA sequence motifs for FOS/JUN family transcription factors. ATAC-seq data suggests these loci are already accessible prior to metal exposure (Figure 4). This supports a model where FOS/JUN are already bound at these sites and MTF-1 affinity for FOS/JUN bound DNA or FOS/JUN proteins is increased in the presence of cadmium. It is also possible that metal could directly facilitate the interaction of MTF-1 and FOS/JUN proteins prior to DNA binding. Alternatively, metal treatment causes a reduction of AP-1 binding, thus increasing the availability of these sites for MTF-1 binding [39]. Future work will be necessary to distinguish between these possibilities.

Cadmium induces gene expression changes and MTF-1 relocalization, but the newly bound MTF-1 regions did not change in chromatin accessibility. This suggests a model whereby MTF-1 is recruited to already accessible regions likely bound by JUN/FOS family transcription factors. This suggests a potential mechanism that cadmium changes the affinity of MTF-1 for FOS/JUN protein–protein interactions or increases affinity for the DNA elements. Since the regions were already accessible, and FOS/JUN sequence motifs were not previously identified for interaction with MTF-1 in the presence of cadmium [35,40], we favor a model for increased protein–protein interactions with AP-1 family transcription factors. Future work will be required to differentiate between these possibilities and how MTF-1 interacts with FOS/JUN or FOS/JUN DNA sequence motifs.

Cadmium induces cell damage and increases AP-1 stress response activity [21,41]. Yang et. al., demonstrated that cadmium treatment in rat cultures accumulated in mid-brain neuron-glia, increasing oxidative stress and activating stress response including AP-1 [42]. Lee et. al., measured increased AP-1 activity in mouse liver induced through cadmium treatment [43]. The AP-1 response to cadmium occurs throughout the body within different organs. Cadmium is detrimental to normal cell growth and development [44] but can also promote oncogenesis and tumor progression [23].

The AP-1 transcription factor was originally found to be oncogenic, but more recent studies have shown that AP-1′s role in tumorigenesis is more complex than initially proposed [45]. Exogenous expression of components of the AP-1 transcription factor proteins containing transactivation domains including c-FOS, FOSB and c-JUN can transform cells [46,47]. Contrary to this, AP-1 transcription factor proteins lacking transactivation domains including JUNB and JUND can act as antagonists of c-Jun [48]. AP-1 regulates both cell proliferation and apoptosis, two potentially opposing forces in tumorigenesis. The specific FOS/JUN protein composition of AP-1 was found to regulate the ability of AP-1 to promote or inhibit tumor growth [49]. Our study demonstrates that cadmium induces MTF-1 binding at FOS/JUN DNA motifs, suggesting a further role in tumor development and one mechanism of how cadmium exposure may transform cells [20]. Future work will be required to investigate the relationship between MTF-1 and FOS/JUN in tumor progression.

The pathways and binding events described here could be part of the cellular defense against stress. AP-1 transcription factors regulate many stress response programs [50]. The specificity of those programs might be dictated by the association with program-specific transcription factors, in this case, MTF-1. In addition to cancer, cadmium disrupts pulmonary and renal functions [51]. This disruption could be through increased cellular ROS induced by cadmium [52], which is mediated through NF-κB and AP-1 signaling [53]. Through interaction with other transcription factors, AP-1 plays a key role in immune response, cellular defense, and in maintaining normal physiology and homeostasis [54]. Therefore, we speculate that MTF-1 in association with AP-1 could facilitate cellular adaptation and stress response to cadmium exposure. Further work would be necessary to understand how MTF-1 and the genes it controls contribute to cellular defense programs and preventing organ toxicity. Understanding these pathways could facilitate diagnosis and treatment following acute cadmium exposures.

MTF-1 is an essential part of cellular metal homeostasis and detoxification. Most heavy metals are toxic and potential carcinogens. Arsenic, cadmium, chromium, and nickel are classified as group 1 carcinogens by the American Cancer Society, and cadmium is found in higher concentrations in breast tumors compared to non-cancerous adjacent tissue [19]. Metals have been correlated with the development and progression of cancers, meaning that our understanding of the metal control pathways are essential to developing new therapeutic avenues to prevent the progression of cancer in humans.

## Data Availability

The data analyzed in this study can be found at the GEO archive (accession numbers: GSE222587, GSE220803 and GSE216496).

## Supplementary Materials

The following supporting information can be downloaded at: https://www.mdpi.com/article/10.3390/cells12060953/s1, Table S1: Differentially expressed genes in cadmium resistant cells; Table S2: Ingenuity pathway analysis upstream regulators.
